# A novel DCC truncating mutation leads to rare congenital mirror movements and corpus callosum agenesis: A case report

**DOI:** 10.1097/MD.0000000000045836

**Published:** 2025-11-14

**Authors:** Gao-Hui Cao, Ai-Qian Zhang, Yi Dong, Liang-Liang Fan, Jian-Yin Yin, Lu-Lu Tang, Ya-Li Li

**Affiliations:** a Department of Cell Biology, School of Life Sciences, Central South University, Changsha, China; b Department of Obstetrics and Gynecology, Third Xiangya Hospital of Central South University, Changsha, China; c Department of Anesthesiology, Hunan Provincial Maternal and Child Health Care Hospital, Changsha, China; d Department of Reproductive Genetics, Hebei Provincial Clinical Medical Research Center for Birth Defects, Hebei General Hospital, Shijiazhuang, China.

**Keywords:** agenesis of corpus callosum, DCC, mirror movements-1, neurodevelopment, whole exome sequencing

## Abstract

**AbstractRationale:**

Mirror movements (MRMVs) are quite common in young children and typically diminish before the age of 10. However, congenital MRMVs often persist into adulthood. MRMV1 is a rare neurodevelopmental disorder characterized by involuntary movements mirroring intentional actions on the opposite side of the body. Recent research has confirmed an intrinsic connection between congenital agenesis of the corpus callosum (ACC) and MRMV1, which is associated with mutations in the deleted in colorectal carcinoma (*DCC*) gene.

**Patient concerns:**

The proband was a fetus, which was prenatally diagnosed with congenital ACC; multiple adult males in the proband’s family were affected by MRMVs.

**Diagnoses:**

The fetus had congenital ACC, and the affected adult males in the family had MRMV1 (congenital mirror movement disorder type 1).

**Interventions:**

Genomic analysis was conducted, including whole-exome sequencing and Sanger sequencing. Given that the proband’s family jointly opted for abortion, no other interventions were employed.

**Outcomes:**

A novel severe mutation in DCC (NM_0005215.3, c.1789C > T/p.Arg597*) was discovered, predicted to be a deleterious mutation through bioinformatics analysis. Sanger sequencing confirmed the segregation of the DCC mutation with the disease phenotype, establishing it as the cause of the familial genetic anomaly.

**Lessons:**

These findings expand the spectrum of DCC mutations associated with MRMV1 and ACC, contributing to genetic counseling and prenatal diagnosis for MRMV patients, and shedding light on the role of DCC in neurodevelopment.

## 1. Introduction

Mirror movements (MRMVs) is a rare neurodevelopmental condition characterized by involuntary movements that mirror and accompany intentional actions on the opposite side of the body.^[1,2]^ These movements typically involve the distal upper limbs and manifest as simultaneous, near-identical reproductions of contralateral voluntary movements.^[3]^ Importantly, while mild MRMVs are frequently observed during the natural developmental course of young children, they tend to spontaneously resolve by approximately 10 years of age.^[1,4]^ In contrast, congenital MRMVs persist into adulthood and are more commonly observed in the upper extremities.^[5]^

At present, there are 4 common types of congenital MRMVs, known as MRMV1 to MRMV4.^[6,7]^ MRMV1 is linked to heterozygous loss-of-function mutations in the deleted in colorectal cancer (*DCC*) gene located on chromosome 18q21 and is often accompanied by agenesis of the corpus callosum (ACC) in some cases.^[8]^ MRMV2 results from mutations in the *RAD51* gene on chromosome 15q15, typically presenting as involuntary MRMVs in the hands and forearms, sometimes causing mild functional impairments. MRMV3 is associated with homozygous mutations in the *DNAL4* gene on chromosome 22q13 and is characterized by MRMVs primarily affecting the hands and fingers, with minimal involvement of proximal limbs. MRMV4, caused by mutations in the *NTN1* gene on chromosome 17p13, is linked to abnormalities in corticospinal tract decussation, leading to difficulties in performing independent unimanual tasks.

The *DCC* gene, situated on chromosome 18q21, spans approximately 1.4 megabytes and consists of 29 exons.^[9,10]^ This gene functions as a receptor for netrin, a pivotal protein responsible for guiding the migration of developing neurons across the midline of the body during embryonic development.^[11–13]^ Mutations within the *DCC* gene have been identified in families affected by MRMVs, further solidifying the association between *DCC* and this neurodevelopmental condition.^[14,15]^ Notably, the intensity of MRMVs in individuals with *DCC* mutations exceeds the typical childhood manifestations and persists into adulthood.^[5]^ Additionally, some patients with *DCC* mutations also exhibit ACC, a congenital absence or underdevelopment of the corpus callosum, further highlighting the complex interplay between genetic factors and neurodevelopmental outcomes.^[16]^ At present, MRMV1 is exclusively attributed to mutations in the *DCC* gene. Furthermore, mutations in the *DCC* gene have been linked to other medical conditions such as colorectal cancer, esophageal carcinoma, familial horizontal gaze palsy with progressive scoliosis type 2, and more.^[17–19]^ This intricate genetic landscape underscores the multifaceted role of the *DCC* gene in both neurodevelopmental processes and the development of various medical conditions.

Here, we studied a Chinese family presenting with Prenatal agenesis of the ACC (proband, Prenatal fetus) and MRMVs-1 (some of the adults in the family). Whole exome sequencing revealed a novel mutation (NM_0005215.3, c.1789C > T/p.Arg597*) in the *DCC* gene within the proband. Sanger sequencing additionally confirmed the presence of this novel mutation in other affected family members, suggesting co-segregation. Furthermore, bioinformatics software predicted that this newly identified *DCC* mutation is deleterious.

## 2. Case presentation

### 2.1. Clinical description

**Figure 1.**
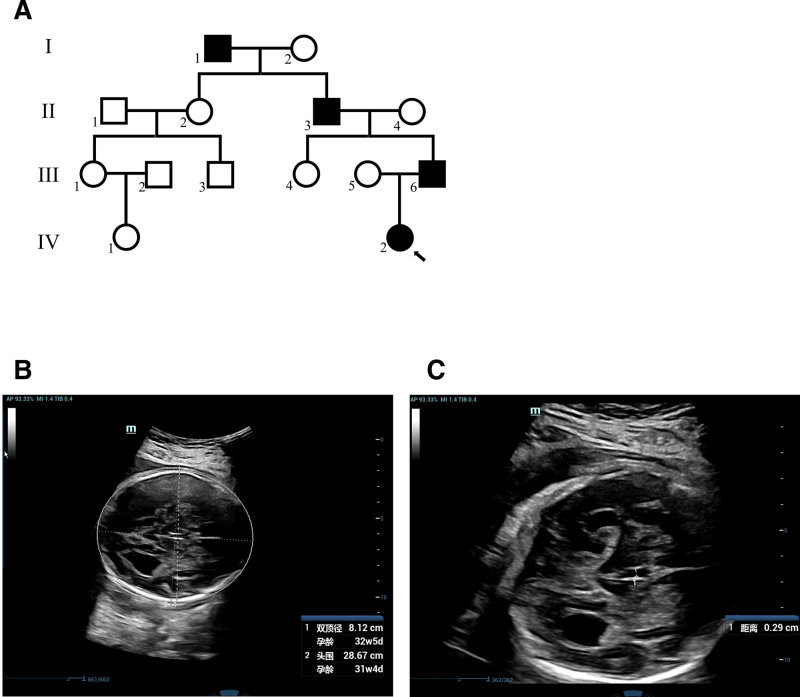
Family map and clinical examination. (A) Family diagram for 4 generations, arrows indicating proband. (B, C) B-ultrasound image of the proband. The examination time was 30 weeks and 4 days of pregnancy.

The proband is a fetus at 30 weeks and 4 days gestational age from Hebei Province, China. The mother is 28 years old with no known abnormalities and is currently pregnant with her first child. Family history investigation reveals that the proband’s grandfather, and great-grandfather all have similar symptoms, that movement and pain are synchronized on both sides of their upper limbs (Fig. 1A). This pregnancy occurred naturally, and there have been no reports of vaginal bleeding or a history of miscarriage in the early stages of pregnancy. Both the nuchal translucency measurement and noninvasive prenatal screening tests indicated low risk. The oral glucose tolerance test results were 4.6–9.5–9 mmol/L. Prenatal ultrasound examination revealed bilateral ventricular enlargement in the proband’s fetal brain, with the left ventricle measuring 10.5 mm and the right ventricle measuring 10.7 mm. The third ventricle measured 3.0 mm, and the septum pellucidum was not clearly visible. Fetal magnetic resonance imaging showed that the midline of the proband’s fetal brain was centered, and the septum pellucidum and corpus callosum were not visible. The father of the proband, who is 28 years old, exhibits MRMVs in the upper limbs. Both lateral ventricles appeared parallel and resembled a teardrop shape (Fig. 1B, C). Given that the families of the proband jointly opted for abortion, no other interventions were employed in this study.

### 2.2. Genetic analysis

Cord blood sampling was performed from proband (IV-2), with a needle inserted into the free section of the umbilical cord to extract 4 mL of fetal blood. Subsequent testing was conducted using the peripheral blood of the parents. Initially, copy number variation-seq technology was used to examine the fetal chromosomes, which did not detect any chromosomal triploid, non-integer chromosomal abnormalities, or deletions or duplications >100k. Then, the proband (IV-2) was selected for whole exome sequencing. BerryGenomics Biotech Company (Beijing, China) conducted exome capture, next-generation sequencing, and standard analysis, including variant annotation referring to Ensemble release 82, and filtering based on ANNOVAR documentation. The data filtering strategies employed were as follows: First, non-synonymous single nucleotide polymorphisms or frameshift-causing insertion-deletion with an alternative allele frequency > 0.05 in the NHLBI Exome Sequencing Project Exome Variant Server (ESP6500), dbSNP152, the 1000 Genomes project, the ExAC database, the genomAD database, and BerryGenomics (an in-house exome database with 2000 exomes) were excluded. Second, filtered single nucleotide variants and insertion-deletion predicted to be damaging by sorting intolerant from tolerant, Polyphen2, and MutationTaster were retained. Third, co-segregation analysis was performed within the family through Sanger sequencing.

**Figure 2.**
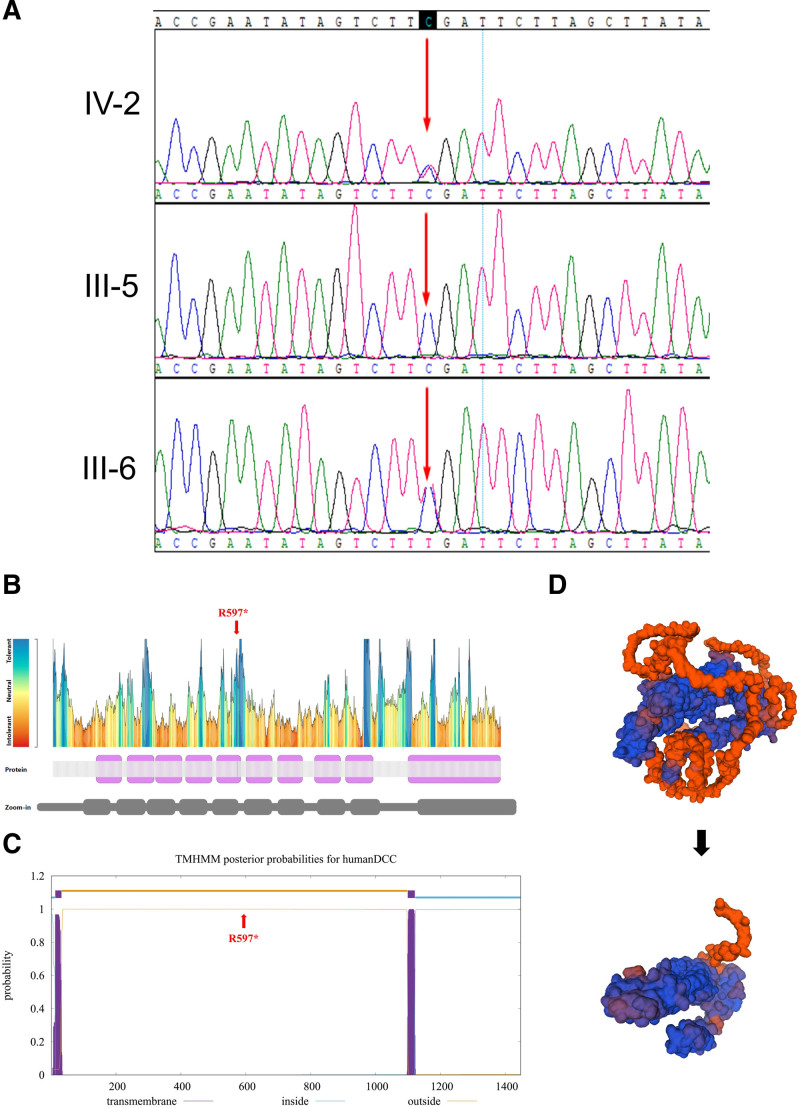
Genetic analysis. (A) Sanger sequencing result. (B) The body is less tolerant to this site of mutation. (C) The mutation results in a complete loss of a transmembrane region of the protein. (D) The top structure represents the native conformation of the DCC protein, while the bottom structure shows the significant conformational disruption caused by the R597* mutation. This truncation leads to a loss of critical domains and alters the overall protein folding. DCC = deleted in colorectal cancer.

The exonic regions of over 20,000 genes, including the mitochondrial genome, were examined. No mitochondrial variations related to the clinical phenotype were detected. A suspected pathogenic potential genetic abnormality was identified in DCC (NM_0005215.3, c.1789C > T/p.Arg597*). The prediction from MutationTaster indicates that this mutation is harmful. There are currently no reported pathogenic associations with this mutation. Sanger sequencing further confirmed the segregation of this mutation with affected family members (Fig. 2A), and it was not found in our control cohort of 200 individuals. This novel mutation results in the termination of Arginine and truncates a portion with numerous conserved sites (Fig. 2B). According to American College of Medical Genetics And Genomics guidelines, this mutation is classified as likely pathogenic (PVS1 + PM2).

## 3. Conclusion

MRMVs were first discovered in cases dating back to 1913.^[20,21]^ In 2020, Sagi-Dain et al reported on a typical family with carriers of a *DCC* mutation, characterized by MRMVs in adult carriers, and prenatal testing revealing corpus callosum underdevelopment and lateral ventricle enlargement in fetal carriers.^[22]^ In vitro studies have indicated that testosterone can influence *DCC* expression, and multiple reports suggest that males may be more sensitive to *DCC* mutations.^[16,23]^ In this study, the proband was a fetus at 30 weeks and 4 days of gestation, with ultrasound examination showing abnormal corpus callosum development and lateral ventricle enlargement. Three of the 4 adult members in the proband’s family were males, suggesting a male bias. Whole exome sequencing and Sanger sequencing further confirmed the *DCC* mutation (NM_0005215.3, c.1789C > T/p.Arg597*) as the genetic factor responsible for the family’s condition. Our research has describe a novel variant of *DCC* mutations and contribute to genetic counseling and prenatal diagnosis for MRMVs patients, offering guidance for family planning.

*DCC* is a single-pass type I membrane protein that consists of 10 domains and 5 regions, with 2 predicted transmembrane segments.^[24,25]^ *DCC is expressed on the surface of spinal cord axons and plays a crucial role in glutamatergic synaptogenesis and plasticity through its interaction with Netrin-1, which mediates signaling essential for these processes.*^[25,26]^ Netrin-1 is a diffusible chemical cue secreted by target cells. Acting as a guidance signal for developing axons, Netrin-1 ensures their accurate navigation to predetermined targets within the nervous system.^[27]^ Therefore, *DCC* plays a crucial role in regulating axonal growth and guidance. Additionally, the structural elements at the N-terminal horseshoe conformation of DCC protein may be necessary for axonal guidance.^[28]^ In our study, a novel mutation in *DCC* (NM_0005215.3, c.1789C > T/p.Arg597*) resulted in the truncation or loss of 5 fibronectin type-III domains and 5 region (Fig. 2B), the loss of 1 transmembrane segment (Fig. 2C), change in protein conformation (Fig. 2D). Interestingly, a previous study reported the p.(Arg597Pro) mutation: a point mutation at position 597 may affect the function of the fibronectin type III-like domain, while the truncating mutation reported in this study would directly lead to the partial loss of this domain and the complete loss of the transmembrane domain.^[29]^ In addition, the truncated region contains several known pathogenic mutations.^[16,29]^ So, this may potentially result in membrane-binding abnormalities and functional impairment of DCC.

Additionally, there are notable clinical differences between bi-allelic and mono-allelic mutations in DCC, with bi-allelic mutations typically leading to more severe pathological outcomes.^[29]^

**Figure 3.**
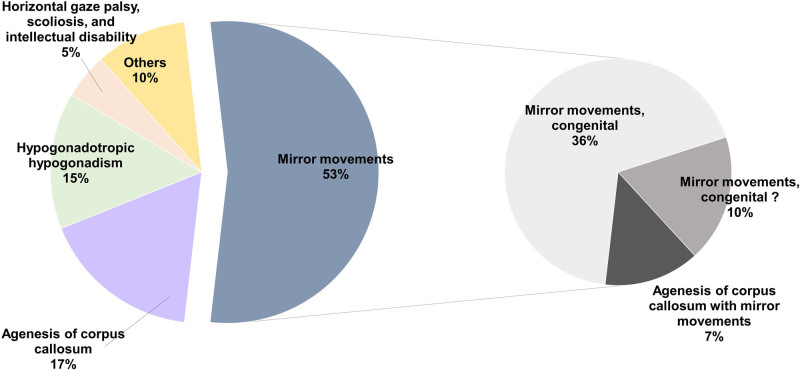
Incidence of each disease in patients with DCC mutations. In the left pie chart, terms such as “Agenesis of corpus callosum,” “Hypogonadotropic hypogonadism,” and “Horizontal gaze palsy, scoliosis, and intellectual disability” represent cases diagnosed with these conditions but without MRMV. The term “others” refers to cases involving DCC mutations that have only been reported in a single instance, such as altered p53 transactivation, ataxia, colorectal cancer, and ovarian cancer. DCC = deleted in colorectal cancer, MRMV = mirror movement.

Currently, a total of 58 *DCC* gene mutations have been reported in patients with various phenotypes. By summarizing these reported DCC mutations (Fig. 3), we observed that the majority of documented *DCC* mutations (53%) manifest as MRMVs, with 7% of carriers reported to have a combination of ACC and MRMVs. In this case, we identified a novel *DCC* mutation (NM_0005215.3, c.1789C > T/p.Arg597*) within a family, resulting in congenital corpus callosum underdevelopment and lateral ventricle enlargement in the prenatal fetus (Fig. 1B, C), while adult family members exhibited MRMVs. This suggests an important role for *DCC* in corpus callosum development during fetal development and contributes to unraveling the link between ACC and MRMV1.

In mice, the loss-of-function mutation in the *DCC* gene results in difficulties maintaining an upright posture, involuntary mirror-like movements in the contralateral limbs, and a gait characterized by skipping during movement.^[30]^ Deactivation of *DCC* in mice leads to axon guidance defects similar to those seen in netrin-1-deficient mice.^[31]^ Furthermore, in *Caenorhabditis elegans*, a *DCC* homolog called UNC40 has been identified.^[32]^ These findings further underscore the significance of this receptor in cross-species neural development.

In summary, our research utilized whole exome sequencing and Sanger sequencing to identify a novel DCC mutation (NM_0005215.3, c.1789C > T/p.Arg597*) in a Chinese family with *DCC*-related disorders. Subsequent analyses confirmed that this mutation is the cause of the genetic anomaly in this family. Our work has broadened the diversity of *DCC* mutations and the recognized list of MRMV1 patients, contributing to a deeper understanding of the relationship between *DCC* and MRMV1.

## Acknowledgments

We are grateful to the patients who agreed to participate in the study for their assistance.

## Author contributions

**Conceptualization:** Ai-Qian Zhang.

**Data curation:** Gao-Hui Cao, Yi Dong, Liang-Liang Fan.

**Formal analysis:** Gao-Hui Cao, Yi Dong, Liang-Liang Fan.

**Funding acquisition:** Liang-Liang Fan, Jian-Yin Yin.

**Investigation:** Ai-Qian Zhang, Jian-Yin Yin.

**Methodology:** Jian-Yin Yin.

**Project administration:** Ya-Li Li.

**Software:** Gao-Hui Cao, Ya-Li Li.

**Supervision:** Lu-Lu Tang.

**Validation:** Lu-Lu Tang.

**Visualization:** Lu-Lu Tang.

**Writing – original draft:** Gao-Hui Cao, Ai-Qian Zhang, Ya-Li Li.

**Writing – review & editing:** Yi Dong, Liang-Liang Fan, Jian-Yin Yin, Lu-Lu Tang.
